# Characterization of mucosa-associated *Escherichia coli* strains isolated from Crohn’s disease patients in Brazil

**DOI:** 10.1186/s12866-020-01856-x

**Published:** 2020-06-23

**Authors:** Rafaella F. A. Costa, Maria L. A. Ferrari, Marie-Agnès Bringer, Arlette Darfeuille-Michaud, Flaviano S. Martins, Nicolas Barnich

**Affiliations:** 1 Departamento de Microbiologia, Instituto de Ciências Biológicas, Universidade Federal de Minas Gerais, Belo Horizonte, Minas Gerais Brazil; 2 Université Clermont Auvergne, Inserm U1071, M2iSH, USC-INRAE 2018, 28 place Henri Dunant, 63000 Clermont-Ferrand, France; 3 Departamento de Clínica Médica, Faculdade de Medicina, Universidade Federal de Minas Gerais, Belo Horizonte, Brazil; 4 Instituto Alfa de Gastroenterologia, Hospital das Clínicas, Universidade Federal de Minas Gerais, Belo Horizonte, Minas Gerais Brazil; 5 Centre des Sciences du Goût et de l’Alimentation, AgroSup Dijon, CNRS, INRAE, Université Bourgogne Franche-Comté, F-21000 Dijon, France

**Keywords:** Crohn’s disease, Inflammatory bowel disease, Mucosa-associated *Escherichia coli*

## Abstract

**Background:**

Crohn’s disease (CD) is characterized by chronic inflammation of the human intestine. Several studies have demonstrated that the intestinal mucosa of CD patients in Western countries is abnormally colonized by adherent-invasive *Escherichia coli* (AIEC) strains. However, no studies to date have focused on the involvement of such *E. coli* strains in CD patients in Brazil. Here, we characterized *E. coli* strains associated with the ileal mucosa of Brazilian CD patients (ileal biopsies from 35 subjects, 24 CD patients and 11 controls).

**Results:**

The colonization level of adherent *Enterobacteriaceae* associated with the ileal mucosa of CD patients was significantly higher than that of the controls. The proportions of *E. coli* strains belonging to phylogroups B1 and B2 were two-fold higher in strains isolated from CD patients than in those isolated from controls. CD patients in the active phase harbored 10-fold more *E. coli* belonging to group B2 than CD patients in remission. Only a few *E. coli* isolates had invasive properties and the ability to survive within macrophages, but 25% of CD patients in Brazil (6/24) harbored at least one *E. coli* strain belonging to the AIEC pathobiont. However, *fimH* sequence analysis showed only a few polymorphisms in the FimH adhesin of strains isolated in this study compared to the FimH adhesin of AIEC collections isolated from European patients.

**Conclusions:**

Mucosa-associated *E. coli* strains colonize the intestinal mucosa of Brazilian CD patients. However, the strains isolated from Brazilian CD patients have probably not yet co-evolved with their hosts and therefore have not fully developed a strong adherent-invasive phenotype. Thus, it will be crucial to follow in the future the emergence and evolution of AIEC pathobionts in the Brazilian population.

## Background

Crohn’s disease (CD) is an inflammatory bowel disease (IBD) characterized by chronic inflammation of the human intestine [1]. The etiology of CD remains unknown, but the most common hypothesis is that chronic inflammation results from an abnormal inflammatory response to intestinal microbiota in a genetically susceptible host [2]. Recent studies have suggested that the composition of intestinal microbiota contributes to CD pathogenesis. In humans, the first evidence of the involvement of intestinal microbiota in IBD came from clinical data showing that diverting the flow of feces relieved the symptoms of CD [3]. Different theories regarding the role of the intestinal microbiota in CD have been developed, in particular the theory of dysbiosis and that of a persistent pathogenic infectious agent in the intestinal mucosa. However, no specific pathogen has yet been definitively identified.

*E. coli* is an important microorganism of the human intestinal microbiota and plays an important role in promoting the stability and maintenance of intestinal physiology. Through the acquisition of virulence factors, some strains of *E. coli* have become pathogenic and are implicated in the etiology of IBD [4]. The search for specific pathogens in the intestinal mucosa of patients with CD has resulted in the identification in 1998 of several candidates, among which Adherent-Invasive *E. coli* (AIEC) have much supporting evidence [5]. Since, a higher prevalence of such AIEC pathobionts, with pro-inflammatory potential, in CD patients compared to healthy subjects, have been reported in various western countries [6–8]. AIEC bacteria behave like pathobiont bacteria rather than real pathogens, and must share in the evolution of common ancestors with ExPEC strains [9]. These bacteria could be the results of an adaptation of non-pathogenic bacteria to environmental factors leading to a phenotypic characteristic. Moreover, this group of bacteria is very heterogeneous (as example AIEC isolated from adults [6, 9–11], children [12, 13], and companion animals [14]) from a phylogenic point of view, which makes it difficult to characterize them at the molecular level. The phenotypic characteristics of these bacteria are: (1) adhesion and invasion of intestinal epithelial cells, involving a process of actin polymerization and recruitment of microtubules [5, 14], and (2) survival and replication in macrophages, leading to the massive release of the cytokine TNF, without inducing the death of the infected macrophage [15]. Two AIEC strains able to adhere to and invade different intestinal epithelial cell lines were isolated from a chronic ileal lesion in a patient with CD (AIEC LF82 (O83:H1) and NRG857c); these strains have been fully identified and closely studied [5, 14]. AIEC are distinct from other pathogenic intestinal *E. coli* in that they do not harbor genes typically associated with other pathogens [14, 16]. Several AIEC genomes were recently sequenced, and comparative genomic studies of *E. coli* strains isolated from patients with CD showed that these strains represent a heterogeneous population with a genomic profile similar to that of extraintestinal pathogenic *E. coli* strains (ExPEC) [17–21]. AIEC colonization in mice leads to strong inflammatory responses in the gut, suggesting that AIEC could play a role in CD immunopathogenesis [10, 11]. Furthermore, the presence of AIEC in the mucosa of CD patients at initial diagnosis suggests that these microorganisms may play a role in the early stages of disease onset [12].

Much of the work on this organism, and its potential role in CD, has been undertaken in Europe, North America and Australia. However, to truly consider the involvement of AIEC in CD, the presence of these bacteria in patients in other parts of the world, including countries with high disease incidence and countries with increasing disease incidence, need to be demonstrated. In the past few decades, the incidence of CD has been increasing in developing countries. No studies have focused on the involvement of such *E. coli* strains in CD patients in Brazil. A recent study detected invasive *E. coli* in the ileum and stools of a CD patient. This strain, while able to adhere to and invade epithelial cells, does not have all the AIEC criteria [22]. The aim of this study was to isolate and characterize *E. coli* strains, particularly AIEC, associated with ileal mucosa in Brazilian CD patients.

## Results

### Patient characteristics

The median ages of in CD patients and healthy controls were 42.3 ± 12.0 years and 57.1 ± 13.4 years, respectively. There were 16 female and 8 male CD patients and 6 women and 5 men in the control group. The ileocolonic area was the most predominantly affected (14 patients: 58.3%), followed by the ileum (9 patients: 37.5%). Colon involvement was present in only one patient (4.1%). Stenosis was observed in 33.3% of patients, penetrating disease in 25.0%, and neither stenosis nor penetrating disease in 20.8%. Patient characteristics are shown in Table 1, patient distribution according to age at CD diagnosis is shown in Table 1, patient distribution according to disease localization is shown in Table 2, and patient distribution according to disease behavior is shown in Table 2.

**Table 1:** Patient characteristics

Patients	N	Gender F/M	Age (Years)	
			Average ± SD	Age range
Control	11	6/5	57.1 ± 13.4	28–81
CD	24	16/8	42.3 ± 12.0	18–66
**Total**	35	22/13	46.1 ± 12.9	18–81

*N* number, *F* female, *M* male, *SD* standard deviation

**Table 2:** Montreal classification of Crohn’s disease

Criteria	n (%)
**Age at Diagnosis (A)**	
A1: 16 years or less	2 (8.3%)
A2: 17–40 years	17 (70.8%)
A3: 40 years or more	5 (20.8%)
**Localization (L)**	
L1: Terminal Ileum	9 (37.5%)
L2: Colon	1 (4.2%)
L3: Ileocolonic	14 (58.3%)
**Behavior (B)**	
B1: Not stenting, non-penetrating	5 (20.8%)
B2: Stenting	8 (33.3%)
B3: Penetrating	6 (25.0%)
B1p: Not stenting, non-penetrating + perianal fistula	1 (4.2%)
B2p: stenting + perianal fistula	2 (8.3%)
B3p: penetrating + perianal fistula	2 (8.3%)

### Mucosa-associated *Enterobacteriaceae* in CD patients and controls

**Fig. 1:**
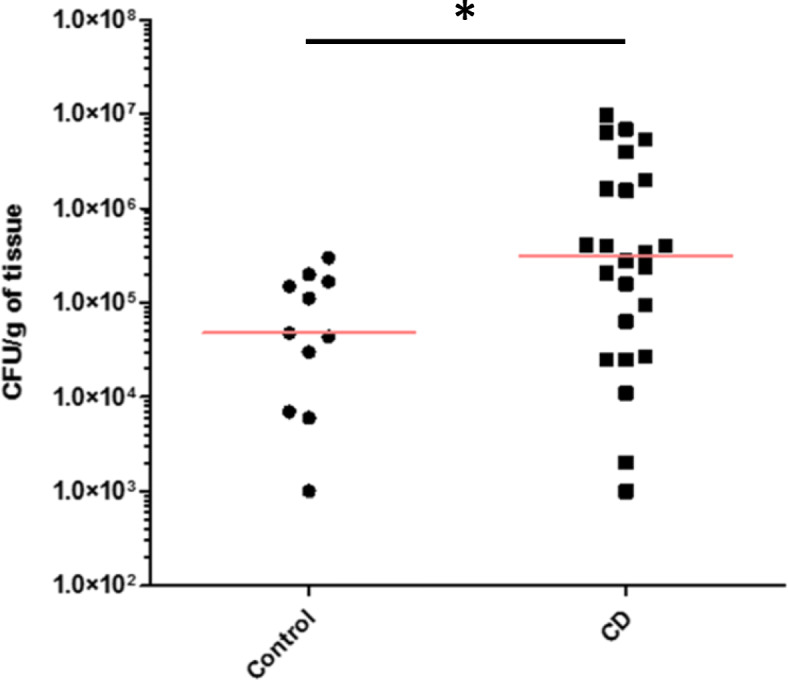
Quantification of *Enterobacteriaceae* associated with the ileal mucosa of controls and patients with CD. The value of the median is shown in red. The statistical analysis was performed using the nonparametric Mann-Whitney test. * *p* < 0.05

Mucosa-associated *E. coli* were isolated from all controls and CD patients. However, a significant 6.45-fold increase (*p* = 0.0252) in the level of mucosa-associated *Enterobacteriaceae* was observed in the ileal mucosa of CD patients (median: 3.1 × 10^5^ CFU/g of tissue) compared with that of controls (median: 4.8 × 10^4^ CFU/g of tissue) (Fig. 1), indicating a higher colonization rate of intestinal mucosa in CD patients than in controls.

Of the 270 *Enterobacteriaceae* isolates obtained in this study, 241 were identified as *E. coli* strains (58 in controls and 183 in CD patients). Concerning the other 29 isolated strains, 10 were *Klebsiella* sp. (2 in control subjects and 8 in CD patients) and 19 were *Enterobacter* sp. (2 in control subjects and 17 in CD patients). All identification was made by nonautomated tests.

### Phylogeny of *E. coli* strains associated with intestinal mucosa

Analysis of the phylogroup distribution of *E. coli* isolated from controls and CD patients showed no significant difference between the two groups (*p* = 0.3797) (Table 3). In the controls, most *E. coli* strains belonged to phylogroups A (44.8%) and D (36.2%). In CD patients, most strains belonged to phylogroups A (37.2%) and B1 (27.9%). The proportions of *E. coli* strains belonging to phylogroups B1 and B2 were two-fold higher in strains isolated from CD patients (B1, 27.9%; B2, 11.5%) than in those isolated from controls (B1, 13.8%; B2, 5.2%). Interestingly, CD patients in the active phase (20.2%) harbored 10-fold more *E. coli* strains belonging to the B2 group than CD patients in remission (2.0%) and 2.4-fold fewer strains of group D (12.8% CD active vs 31.3% CD remission).

**Table 3:** Distribution of *E. coli* strains among the phylogenetic groups

Patients	Strains	Phylogenetic groups^**a**^			
		A	B1	B2	D
Control	58	26 (44.83%)	8 (13.79%)	3 (5.17%)	21 (36.21%)
CD	183	68 (37.16%)	51 (27.87%)	21 (11.48%)	43 (23.50%)

^a^The statistical analysis was performed by the Kruskal-Wallis test adjusted by Dunn’s test. No statistical difference was observed

### Ability of *E. coli* strains to invade human intestinal epithelial cells

**Fig. 2:**
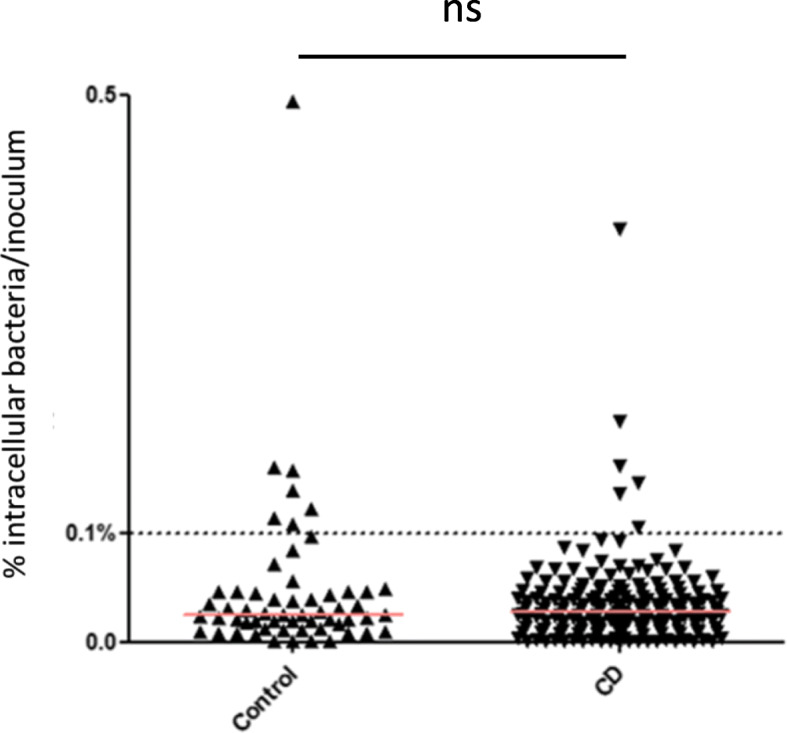
Invasive ability of *E. coli* strains. The results are expressed as the percentage of inoculum surviving in human Intestine-407 epithelial intestinal cells after 3 h of infection and 1 h of gentamicin treatment. The value of the median is shown in red. The statistical analysis was performed using the nonparametric Mann-Whitney test. n.s. *p* > 0.05

We determined the ability of the 241 *E. coli* strains isolated in this study to invade human intestinal epithelial I-407 cells. Intracellular bacteria with an abundance equal to or greater than 0.1% compared to the inoculum were classified as invasive to I-407 cells [23]. Of the 241 *E. coli* strains isolated from intestinal mucosa, 13 (5.4%) were considered invasive to epithelial cells, 7 strains in the control group and 6 in CD patients (Fig. 2). There was no global significant difference between the cell invasion profile of strains isolated from the mucosa of the control and CD groups (*p* = 0.9225). Assessment of controls and CD patients showed that the abundances of most intestinal-associated *E. coli* strains (94.6%) were lower than 0.1%, indicating that most strains isolated in this study had not yet evolved to select the ability to invade intestinal epithelial cells.

### Survival and replication within THP-1 cells

**Fig. 3:**
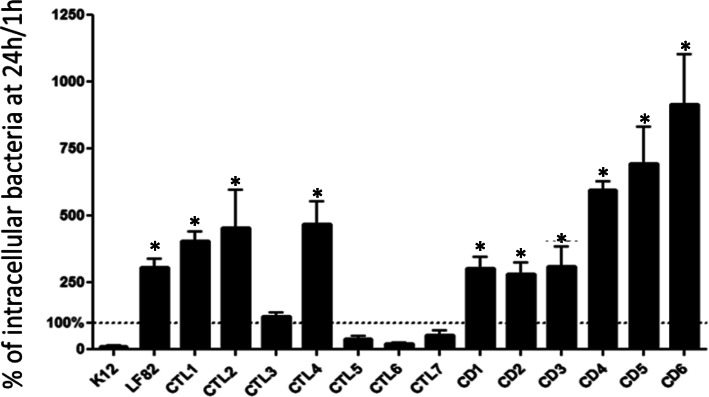
Survival and replication of *E. coli* strains within THP-1 cells. CTL, control subject samples; CD, CD patient samples. The statistical analysis was performed using a nonparametric Mann-Whitney test to determine if the value corresponding to the number of intracellular bacteria at 6 h post-infection was significantly higher than the value at 1 h post infection for each strain. * *p* < 0.05

**Fig. 4:**
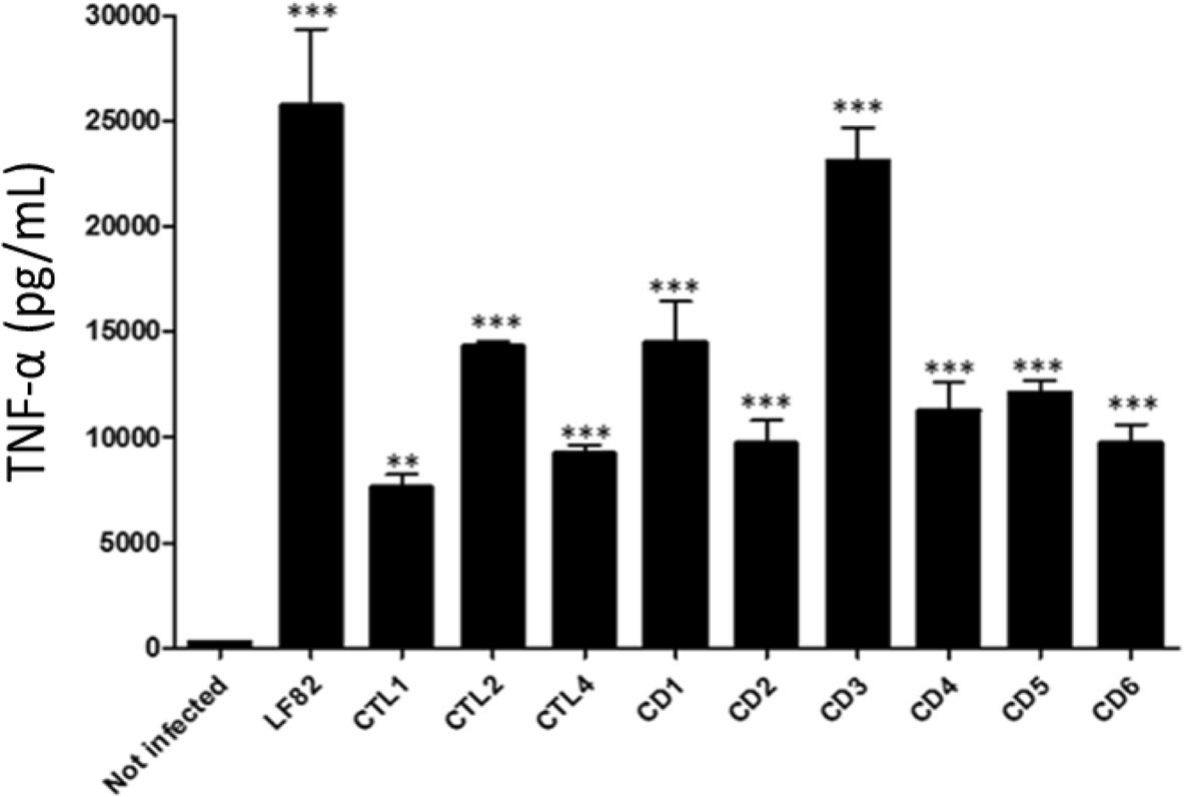
TNF-α levels produced by THP-1 cells infected with AIEC strains. CTL, control subject samples; CD, CD patient samples. Statistical analysis was performed by one-way ANOVA followed by the Newman-Keuls test. ** *p* < 0.01, *** *p* < 0.001 (compared to noninfected cells)

All 13 *E. coli* strains considered invasive to I-407 cells were used for intracellular replication assays in human THP-1 macrophages. Assay results with values significantly higher than 100% (percentage of the number of bacteria at 6 h post-infection compared to 1 h post-infection) indicated that the strains were able to replicate within THP-1 cells after 24 h of infection. Of the strains tested in this study, 9 *E. coli* strains (3 in the control group and 6 in CD patients) were able to multiply within macrophages (Fig. 3). As these 9 *E. coli* strains were invasive to I-407 cells and were able to replicate within THP-1 cells, they were classified as AIEC. The ability of two out of 9 AIEC strains isolated from CD patients (samples CD5 and CD6) to survive in THP-1 cells was significantly high compared to the reference AIEC strain LF82. The production of TNF-α in THP-1 cells infected with the 9 *E. coli* strains identified as AIEC was measured. The strain LF82 was used as a positive control, and *E. coli* K-12, which is unable to replicate in macrophages, was used as a negative control. TNF-α levels were significantly higher in cells infected with AIEC strains than in uninfected cells (Fig. 4). There was no significant difference in TNF-α levels between AIEC strains isolated from controls and CD patients. In conclusion, 6 out of 24 CD patients harbor at least one AIEC strain (25%) compared to 3 out of 11 controls (27%).

### Association between AIEC profile and the presence of putative virulence factors and/or *fimH* polymorphisms

We first looked for the presence of the *hcp*1 and *hcp*3 genes, one of the genes of the type VI secretion system potentially involved in AIEC virulence, and the *lfp*A and *gip*A genes, involved in the ability of AIEC to interact with Peyer’s patches, in AIEC strains isolated from Brazilian CD patients. We observed no association between the presence of these four genes and the AIEC phenotype of AIEC strains isolated from Brazilian CD patients (Table 4).

**Table 4:** Analysis of the presence of putative virulence factors in AIEC strains

Strains	Phylogroup	***hcp***1	***hcp***3	***lpf***A	***gip***A
LF82	B2	+	+	+	+
CTL1	B2	–	+	–	–
CTL2	D	+	–	–	–
CTL4	A	–	–	–	–
CD1	B1	–	–	+	–
CD2	B1	–	–	+	–
CD3	B2	–	–	–	–
CD4	B1	–	–	–	–
CD5	B1	–	–	–	–
CD6	B1	–	–	–	–

In addition, the *fim*H gene in all AIEC strains isolated in this study was sequenced. For comparison, the *fim*H sequences of the LF82 and K12 strains were used for the alignment of the *fim*H sequences. In the 9 AIEC strains, 43 single base polymorphisms (SNPs) were identified in the *fim*H gene, resulting in the substitution of eight amino acids in the protein sequence. Of the 9 AIEC strains, 8 had at least one amino acid polymorphism, and only 1 (isolated from the control group, CTL2) had 100% similarity compared to the *fim*H gene of *E. coli* K12 (Table 5). Only the V27A mutation was observed in the strains isolated in this study. As reported elsewhere [24], the V27A polymorphism is not found exclusively in AIEC strains. Thus, this variation is not classified as an exclusive pathoadaptive change associated with the AIEC phenotype but could be a mark of the transition from commensalism to pathobionts in *E. coli*.

**Table 5:** Substitutions of amino acid residues in the AIEC strains

Strains	Mutations at amino acid residue									
	Leader Lectin domain Pilin domain peptide									
	6	26	27	66	70	78	119	158	202	269
**K12**	**T**	**P**	**V**	**G**	**N**	**S**	**A**	**T**	**A**	**Q**
LF82			A		S	N		P		
CTL1			A							K
CTL2										
CTL4			A	S					V	
DC1		L	A							
DC2		L	A							
DC3			A		H					
DC4			A							
DC5			A							
DC6			A							

## Discussion

The intestinal microbiota has been associated with the etiopathogenesis of IBD [25]. Changes in the composition of the enteric microbial community, with a decrease in the number of resident bacterial species and an increase in the number of bacteria associated with the mucosa, have been observed in CD. Several independent studies have shown a considerable increase in the population of *Enterobacteriaceae*, especially *E. coli*, in the intestinal mucosa of CD patients compared to control subjects [26, 27]. Several studies have demonstrated that the intestinal mucosa of CD patients in Western countries is abnormally colonized by pathobiont *Escherichia coli* strains with adherent and invasive properties (AIEC) [5–13, 23, 28]. Much of the work on AIEC, as well as their potential role in CD, has been undertaken in areas of high CD incidence (Europe, North America and Australia). It was currently unclear whether the changes in such putative pathogen organisms identified in Western populations are more widespread in IBD patients in regions with increasing IBD incidence, especially in Latin America and more specifically in Brazil.

Phylogenetic analyses have shown that *E. coli* strains are classified into four main phylogenetic groups (A, B1, B2 and D). Here, we demonstrate that the mucosa of Brazilian CD patients is also heavily colonized by *Enterobacteriaceae*, mostly by *E. coli* strains. The strains isolated in this study have a more homogeneous profile when classified according to the major phylogenetic groups. However, we observed a considerable increase in the phylogroups B1 and B2 in Brazilian CD patients compared to controls, and the CD patients in the active phase harbored 10-fold more *E. coli* belonging to the B2 group than CD patients in remission. The *E. coli* strains belonging to groups A and B1 are weakly or nonpathogenic, while those belonging to groups B2 and D are most often pathogenic and are commonly associated with urinary tract and extraintestinal infections [29–32]. *E. coli* strains isolated from IBD patients in Western countries are mostly classified into groups B2 and D. While some researchers observed a similar distribution of phylogenetic groups in the control and IBD groups [13, 33–38], other studies, in agreement with our data, have reported increased colonization by strains belonging to groups B2 and D in IBD patients compared to controls [10, 24].

Several factors of CD predispose patients to the emergence of an intestinal microenvironment advantageous for effective colonization by *E. coli*, thereby contributing to susceptibility to the disease. As stated previously, *E. coli* is an important microorganism of the normal intestinal microbiota. However, *E. coli* strains associated with the mucosa of CD patients possess unusual properties, such as the ability to adhere to and invade the intestinal epithelium and to multiply within macrophages, leading to the production of large amounts of TNF-α [5, 14, 15, 23]. The results of our study show that most *E. coli* strains isolated in both CD patients and controls did not have the invasive phenotype. However, 6 out of 24 CD patients harbored at least one strain with characteristics of AIEC. This prevalence of CD patients harboring AIEC bacteria is consistent with data reported in the literature of adult cohorts [39]. In contrast, the prevalence of AIEC bacteria in the controls in this study was abnormally high compared to the literature. This can be explained by the low number of controls included (*n* = 11) and by the fact that the controls were hospitalized patients without IBD but with other comorbidities. The 3 control patients harboring AIEC bacteria had schistosomiasis (CTL1), gallbladder neoplasia in 2009 and cholecystectomy in 2009 (CTL2), and Still’s disease-rheumatic fever (CTL3). However, several independent studies have demonstrated that the mucosa of CD patients is heavily colonized by AIEC strains in comparison to a control group. The reasons for this discrepancy are not fully understood. However, it is believed that the presence of AIEC associated with the mucosa of CD patients can vary according to different populations. One of the hypotheses to explain the high prevalence of AIEC in Western countries is environmental exposure over a long period. It is likely that over the next few decades, *E. coli* with invasive properties will be selected in the ileal mucosa of Brazilian CD patients with abnormal colonization of adherent *E. coli* as Brazilian CD patients gradually adopt a more Western lifestyle; alternatively, the ileal mucosa of Brazilian CD patients could be contaminated by more aggressive AIEC pathobiont strains whose circulation in such populations would be favored by global travel.

Despite all the studies that have assessed the pathogenicity of AIEC strains, the genetic factors leading to the AIEC phenotype have not yet been fully characterized. AIEC strains have high genome variability, complicating the identification of specific genetic factors of the pathotype [27]. The FimH adhesin plays an essential role in the virulence of AIEC strains; bacterial adhesion occurs via an interaction between FimH adhesion and mannosylated CEACAM6 adhesion molecules, which are abnormally expressed in the ileal mucosa of 35% of CD patients in Europe [40]. Through its lectin domain, the FimH protein mediates bacterial adhesion to mannose residues present in the CEACAM6 receptor [41]. The presence of protein polymorphisms resulting from substitutions in the amino acid sequences of some FimH proteins is correlated with an increase in the ability of AIEC to adhere to the intestinal epithelium, increasing the affinity of FimH to mannose residues exposed by the CEACAM6 receptor [24]. The presence of FimH polymorphisms is thought to be one of the virulence evolution mechanisms of AIEC pathogenicity that leads to the development of chronic inflammatory bowel disease in genetically susceptible hosts. In a mouse model, it was reported that mutated FimH variants increased the ability of urinary *E. coli* isolates to colonize the urinary tract, and these variants were correlated with the overall extraintestinal virulence of *E. coli* [42, 43]. As noted in this study, significant variations of the FimH protein of the AIEC strains isolated from Brazilian CD patients and controls have not yet been selected, except for the A27V polymorphism. Even if we showed that A27V is found in most natural FimH variants, V27A is unlikely to be pathoadaptive for AIEC in CD, as it is not sufficient to mediate a high level of bacteria binding to mannosylated CEACAM6 [24]. Another study argued that the V27A substitution (i.e., with alanine in position 27) is the primary pathoadaptative FimH mutation arising in AIEC isolated from CD pediatric patients in the entire spectrum of mucosal inflammation [44].

Other AIEC bacterial factors play a role in AIEC virulence. This is the case for the *lpf*A and *gip*A genes, which participate in the processes of AIEC interaction and survival in Peyer’s patches via M cell interaction [45, 46]. The type 6 secretion system (T6SS) seems to be strongly involved in AIEC pathogenicity, but the means by which the secretion system contributes to AIEC strain virulence remain to be elucidated. The low prevalence of these genes in the *E. coli* collection isolated in this study suggests that these mucosa-associated *E. coli* strains do not have a highly pathogenic profile. Furthermore, in agreement with the results presented above, the few strains isolated from Brazilian subjects and characterized as AIEC lack the virulence genes of the AIEC strain LF82. Note that in contrast to findings in other reports [10, 24], the AIEC strains isolated in this study belong mainly to the B1 phylogroup. These discrepancies suggest that the profile of AIEC differs according to the population in which they are isolated and that environmental, nutritional and genetic factors may be correlated both with susceptibility to disease and the success of colonization of the intestinal mucosa by strains of pathogenic *E. coli*. In our study, only a few *E. coli* isolates from Brazilian subjects had invasive properties and the ability to replicate within macrophages. In addition, sequence analysis of *fim*H genes of *E. coli* strains showed no selection of significant *fim*H polymorphisms associated with virulence, unlike in AIEC collections isolated from European patients. This indicates that strains isolated in Brazilian CD patients have probably not yet co-evolved to develop a strong adherent-invasive phenotype. We can hypothesize that these B1 and B2 strains selected in Brazilian CD patients will evolve in the future. Thus, it will be of great interest to follow the evolution of the pathogenicity of these strains over the next decade with the aim of gaining a better understanding of the high prevalence of AIEC in developed countries.

## Conclusions

In our study, we have shown that *E. coli* strains colonize the intestinal mucosa of Brazilian CD patients; however, the strains isolated from Brazilian CD patients have probably not yet co-evolved with their hosts and therefore have not developed a strong adherent-invasive phenotype. Thus, it will be important to conduct further studies to investigate the evolution of such strains in the Brazilian population to understand the emergence of AIEC pathogens in Western countries or to follow the circulation of the more aggressive AIEC pathobiont strains in Brazilian populations, as these strains could be favored by global travel.

## Methods

### Patients

Thirty-five subjects, 24 CD patients and 11 controls, were enrolled between March 2012 and August 2013 at the Instituto Alfa de Gastroenterologia (IAG), Hospital das Clínicas (HC), Federal University of Minas Gerais (UFMG), Belo Horizonte, Brazil. The diagnosis of CD in the patients was based on clinical, laboratory, radiological, endoscopic and histopathological findings. The control group comprised individuals who had undergone routine ileocolonoscopy at the hospital without any clinicopathological diagnosis of inflammatory disease. None of the patients had received antibiotics in the 2 weeks before sampling. Ileal mucosa biopsy specimens were spread onto MacConkey agar, and the isolates were identified as previously described [47]. The strains were isolated in Brazil and characterized by a metabolic macromethod using an old procedure used in the article published by Pessoa et al. [47]. The characterization of the strains was then confirmed in the laboratory in France, following isolation on Drigalski selective medium. Then, strain identification was confirmed using the well-established API 20 E strips (Biomerieux) method for manual microorganism identification at the species level, and APIWEB software containing all of the API databases was used for a reliable automated interpretation of API strip results.

### Bacterial strains

Of the Enterobacteriaceae isolated from mucosa, 241 were identified as *E. coli* strains, 183 from the mucosa of CD patients and 58 from controls. The AIEC reference strain LF82, which was isolated from a chronic ileal lesion of a CD patient [5], and the nonpathogenic *E. coli* K-12 strain MG1655 were used in the experiments. Bacteria were grown in Luria-Bertani (LB) broth overnight at 37 °C without shaking.

### *E. coli* phylotyping

The *E. coli* phylogroup of each strain was determined by the multiplex PCR technique described by Clermont et al.; this technique assigns strains to phylogroups A, B1, B2 and D based on the presence/absence of three genes (*chuA*, *yjaA* and *TspE4.C2*) [48].

### Cell lines and cell culture

Human intestinal epithelial Intestine-407 cells (ATCC, CCL-6) were maintained in an atmosphere containing 5% CO_2_ at 37 °C in modified Eagle’s Minimal Essential Medium (EMEM, PAA) supplemented with 10% fetal bovine serum (FBS; Lonza), 1% nonessential amino acids (PAA), 1% L-glutamine (PAA), 200 U/L penicillin, 50 mg/mL streptomycin and 0.25 mg/L amphotericin B with 1% vitamins (PAA).

Human monocytic THP-1 cells (ATCC, TIB-202) were maintained in an atmosphere containing 5% CO_2_ at 37 °C in RPMI 1640 medium (PAA) supplemented with 10% FBS and 1% L-glutamine (PAA). THP-1 monocytes were differentiated into macrophages by treatment with 20 ng/mL phorbol myristate acetate (PMA; Sigma) for 24 h.

### Invasion assay

The number of invasive bacteria in Intestine-407 cells was determined by the gentamicin protection assay as previously described [14]. Briefly, Intestine-407 cells were seeded at a density of 2 × 10^5^ cells/cm^2^ in 24-well plastic plates in the cell culture medium described above without antibiotics. After 24 h of culture at 37 °C and 5% CO_2_ (allowing cells to reach 80–90% confluence), the cells were infected at a multiplicity of infection (MOI) of 10 for 3 h. Cells were washed with PBS and incubated in cell culture medium containing 100 μg/mL gentamycin (Euromedex) for 1 h to kill extracellular bacteria (the bactericidal effect of gentamicin at this concentration was validated on all the strains tested). After washing, the epithelial cells were lysed with 1% Triton X-100 (Sigma) in deionized water. Samples were diluted and plated onto LB agar plates to determine the number of CFUs 24 h later.

### Macrophage survival assay

THP-1 cells were seeded on 24-well tissue culture plates at a density of 2.5 × 10^5^ cells/cm^2^ and infected at an MOI of 25 for 20 min. Infected cells were then washed and incubated with culture medium containing 50 μg/mL gentamicin for 40 min (1 h after infection) or 24 h (24 h after infection) [49].

### Enzyme-linked immunosorbent assay for TNF-α quantification

Macrophages were infected as described above, and the amount of TNF-α in the culture supernatant was measured. All collected supernatants were frozen at − 80 °C until processing. Released pro-inflammatory TNF-α cytokines were quantified in the supernatant by ELISA using kits from R&D systems following the manufacturer’s instructions.

### Detection of virulence factors by PCR

The presence or absence of the *hcp*1, *hcp*3, *lpf*A and *gip*A genes was analyzed by PCR. The primers, PCR conditions and product sizes are given in Table 6.

**Table 6:** Primer sequences and PCR conditions used to detect the *hcp*1, *hcp*3, *lpf*A, *gip*A and *fim*H genes

Gene	Primer sequencing (5’- 3′)	PCR conditions	Product (pb)	Reference
*hcp*1	F: AAACACCACTGGAGTACCTG R: TGGTACTTAGCAAGAAAGAGC	95 °C 3 min., 35 cycles (95 °C 45 s., 55 °C 30 s, e 72 °C 45 s.) e 68 °C 8 min.	999	This study
*hcp*3	F: CGAAATCAGTCTTGTTCCGC R: GGGTTTCCTTATCGTGTTCT	95 °C 3 min., 35 cycles (95 °C 45 s., 55 °C 30 s, e 72 °C 45 s.) e 68 °C 8 min.	745	This study
*lpf*A	F: GGCCTTCTTTCAGACGGTA R: CTGGAAAACTGCGATATCTCC	95 °C 3 min., 35 cycles (95 °C 45 s., 57 °C 30 s, e 72 °C 45 s.) e 68 °C 8 min.	199	[45]
*gip*A	F: GTCGTTGCGCCACCAACAA R: ACGGCGCAGATGGTAATTCT	94 °C 5 min., 35 cycles (94 °C 1 m., 55 °C 1 m, e 72 °C 30 s.) e 72 °C 10 min.	200	[46]
*fim*H	F: ATTCCTCACAATCAGCGCAC R: CGCGTCTTATCTGGCCTACA	95 °C 3 min., 35 cycles (95 °C 45 s., 57 °C 30 s, e 72 °C 45 s.) e 68 °C 8 min.	1125	This study

### Sequencing of the *fimH* gene

To amplify the *fim*H gene, a pair of primers was designed based on conserved regions of the *E. coli* sequences deposited in GenBank: K-12 MG1655 (NC000913.3), LF82 (AF288194.1), UPEC (NC004431) and APEC 01 (NC008563). Primers and PCR conditions are given in Table 6. After purification with the Wizard_SV Gel and PCR Clean-Up System (Promega, Madison, WI, USA), PCR products were sequenced (Sanger sequencing technique using the barcode system, Eurofins Genomics Europe Sequencing, Germany). Nucleotide sequences were obtained from both strands and analyzed for editing by the CAP3 Sequence Assembly Program (http://pbil.univ-lyon1.fr/cap3.php) and the BLAST homology search program (blastN) (http://www.ncbi.nlm.nih.gov/BLAST/). Alignment analysis was carried out by the Multalin Page interface program (http://multalin.toulouse.inra.fr/multalin). The sequences were converted into amino acids with the Expasy Translate Tool (http://ca.expasy.org/tools/dna.html).

### Statistical analysis

The data were analyzed with GraphPad Prism software version 5.00 (GraphPad Software, San Diego, California, USA). To compare proportions of strains belonging to different phylogroups, we carried out statistical analysis using the Kruskal-Wallis test adjusted by Dunn’s test. To compare the values of bacteria associated with the ileal mucosa or the level of invasion of different isolated bacteria between CD patients and controls, since the values do not present a normal distribution, we used the nonparametric Mann-Whitney test. For each strain, to determine the value corresponding to the number of intramacrophagic bacteria at 24 post-infection was significantly higher than that at 1 h post-infection, we used the nonparametric Mann-Whitney test. To compare the level of TNF alpha secretion by macrophages infected with AIEC strains at 24 h post-infection compared to uninfected cells, we used a one-way ANOVA test followed by the Newman-Keuls test. All experiments were performed at least three times. The results with *p* < 0.05 were considered significant.

## Data Availability

All data generated or analyzed during this study are included in this article.

## Abbreviations

CD: Crohn’s disease
AIEC: Adherent-invasive *Escherichia coli*
IBD: Inflammatory bowel disease
ExPEC: Extraintestinal pathogenic *E. coli* strains
T6SS: Type 6 secretion system
IAG: Instituto Alfa de Gastroenterologia
HC: Hospital das Clínicas
UFMG: Federal University of Minas Gerais
LB: Luria-Bertani
PCR: Polymerase chain reaction
MOI: Multiplicity of infection
SNPs: Single base polymorphisms
